# Adverse food reactions and alterations in nutritional status in children with autism spectrum disorders: results of the NAFRA project

**DOI:** 10.1186/s13052-024-01794-8

**Published:** 2024-11-04

**Authors:** Serena Coppola, Rita Nocerino, Franca Oglio, Paola Golia, Maria Candida Falco, Maria Pia Riccio, Laura Carucci, Teresa Rea, Silvio Simeone, Raffaele Garotti, Nadia Marani, Carmela Bravaccio, Roberto Berni Canani

**Affiliations:** 1 Department of Translational Medical Science, University of Naples “Federico II”, Via S. Pansini 5, Naples, 80131 Italy; 2 CEINGE-Advanced Biotechnologies, ImmunoNutritionLab, University of Naples “Federico II”, Naples, Italy; 3 Department of Biomedicine and Prevention, University of Rome “Tor Vergata”, Rome, Italy; 4 Department of Maternal and Child Health, Child and Adolescent Psychiatry, AOU “Federico II”, Naples, Italy; 5 Department of Public Health, University of Federico II, Naples, Italy; 6 Department of Clinical and Experimental Medicine, University of Catanzaro Magna Graecia, Catanzaro, Italy; 7 Task Force on Microbiome Studies, University of Naples “Federico II”, Naples, Italy; 8 European Laboratory for the Investigation of Food-Induced Diseases, University of Naples “Federico II”, Naples, Italy

**Keywords:** Food allergy, Celiac disease, Food intolerance, Malnutrition, Mediterranean diet, Obesity, Food selectivity

## Abstract

**Background:**

To assess the adverse food reactions (AFR) prevalence in children with autism spectrum disorder (ASD) and in non-ASD healthy controls (NASD). Nutritional status alterations, food selectivity and adherence to Mediterranean Diet (MD) were also evaluated.

**Methods:**

The NAFRA (Nutritional status and Adverse Food Reactions in children with Autism Spectrum Disorder) project was an observational, case-control, comparative study conducted at a tertriary center for pediatrics involving Caucasian patients of both sexes, aged 18 months-7 years, with a diagnosis of ASD, and matched NASD controls.

**Results:**

From October 2017 to December 2023, 100 ASD patients [79 male, mean (± SD) age 49.9 months (± 15.4)] and 100 NASD controls [75 male, mean (± SD) age 49.8 months (± 17.7)] were enrolled at the Pediatric Section of the Department of Translational Medical Science of the University of Naples Federico II. A significantly higher prevalence of AFR was observed in ASD patients if compared with NASD (16% vs. 2%, *p* = 0.001), mainly due to a higher prevalence of food allergy (7% vs. 1%, *p* = 0.03). A significantly higher prevalence of food intolerance and celiac disease was also observed in ASD children. The rate of obesity was significantly higher in ASD patients compared to NASD. Food selectivity and low MD-adherence were more frequent in ASD children (26% vs. 2%, *p* < 0.0001 and 28% vs. 16%, *p* = 0.041, respectively).

**Conclusions:**

The high rate of AFR, obesity and unhealthy dietary habits observed in ASD children strongly suggest the importance of a multidisciplinary approach, providing early diagnosis of AFR and appropriate nutritional management to improve core and associated ASD-related conditions.

**Trial registration:**

The NAFRA Project was registered on https://clinicaltrials.gov/ with the identifier NCT04719923. Registered 18 January 2021. https://clinicaltrials.gov/study/NCT04719923.

**Supplementary Information:**

The online version contains supplementary material available at 10.1186/s13052-024-01794-8.

## Introduction

Autism Spectrum Disorder (ASD) is an early-onset complex and heterogeneous neurodevelopmental group of conditions, which present as main characteristics deficit in social communication and interaction, together with restrictive and repetitive behaviors, interests, or activities, and with symptoms severity that varies widely among patients [1]. In the last decades, ASD diagnoses have doubled: to date, 1/100 children at worldwide level, and 1/77 in Italian children has ASD, with a ratio of males to females quoted as 4:1 [2, 3]. ASD has life-time consequences with negative impacts on overall health, social integration, and quality of life [4]. Furthermore, the economic burden for health care systems and families is very high including direct and indirect costs for therapies, specialized educational requirements and support, and family/caregivers’ loss of productivity [5].

The ASD often co-exists with a range of comorbidities, encompassing both physical and mental health disorders [6, 7]. Evidence suggested that children with ASD could present also an increased risk for developing adverse food reactions (AFR), such as food allergies/intolerances and celiac disease (CD) [8]. Unfortunately, the limitations related to the study design, the low sample size, the diagnostic procedures adopted for AFR diagnosis (e.g., parental-reported diagnosis, or the use of inaccurate and a-specific diagnostic procedures), and the overall quality of the studies strongly suggest the importance of more research on the prevalence of AFR in ASD children [9–21].

Furthermore, it has been suggested that ASD pediatric patients could present also an increased risk of malnutrition [22–26], potentially deriving at least in part by restrictive and repetitive dietary habits, presence of other comorbidities, hyperactivity, parental nutritional beliefs [27–29]. This could negatively impact the diet quality, which is characterized by increased exposure to energy-dense ultra-processed foods and sweet beverages and by low adherence to the Mediterranean Diet (MD) key foods, such as fruits, vegetables, legumes and whole grains [30–33]. Altogether, these feeding problems may facilitate the occurrence of nutritional status alterations and of immune-mediated/inflammatory disorders [25, 34–38]. These conditions may further contribute to the behaviors severity and to worsen the daily functioning, social interactions, and the overall quality of life of the ASD patients [39, 40].

Understanding the intricate interplay between dietary habits and AFR susceptibility and nutritional status alterations in ASD patients may hold promise for innovative preventive and therapeutic nutritional interventions. Providing appropriate nutritional management by a multidisciplinary team could have a pivotal role in the early phase of ASD management for preventing and treating relevant co-morbidities in these children and to improve core and associated ASD-related conditions.

The NAFRA (*N*utritional status and *A*dverse *F*ood *R*eactions in children with *A*utism Spectrum Disorder) project was designed to firstly evaluate the AFR prevalence in ASD pediatric patients and in non-ASD healthy controls (NASD). Nutritional status alterations, food selectivity and adherence to the MD were also evaluated. Further evaluation included the analysis of gut microbiome structure and function, as potential underlying action mechanisms in the AFR pathogenesis.

In this paper, we provided the results of prevalence of AFR and alterations in nutritional status in children with ASD.

## Methods

### Study design and ethics

**Fig. 1:**
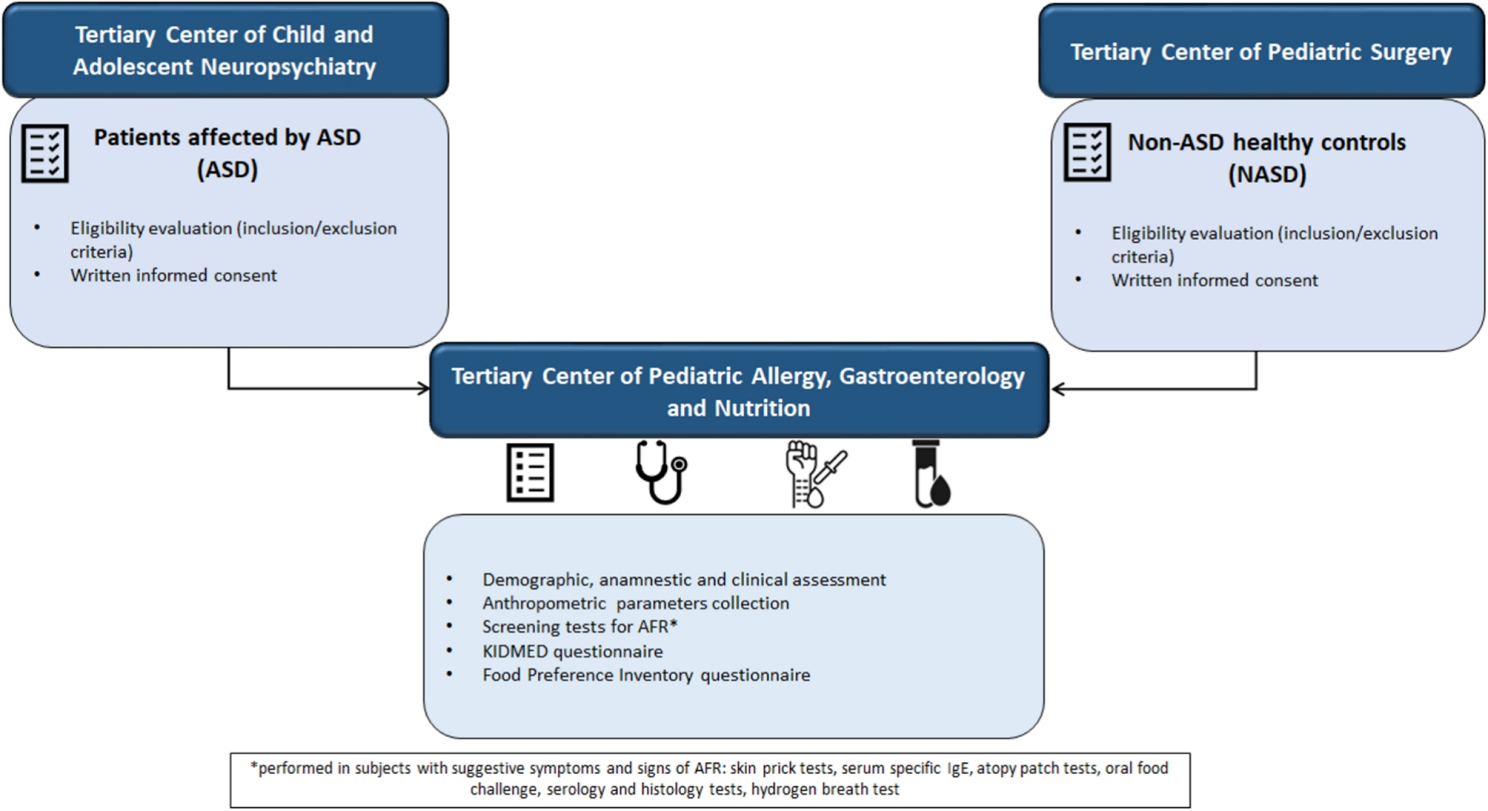
Graphic representation of the study design. AFR, adverse food reactions. Figure shows graphically the design of the NAFRA study

The NAFRA Project was an observational, case-control, single-center study conducted from October 2017 to December 2023 at the Pediatric Section of the Department of Translational Medical Science of the University of Naples Federico II. The recruitment of ASD patients took place at the Tertiary Center of Child and Adolescent Neuropsychiatry, whereas the NASD healthy controls were recruited at the Tertiary Center of Pediatric Surgery. After the enrollment, all study subjects were also assessed at the Tertiary Center of Pediatric Allergy, Gastroenterology and Nutrition. The study was approved by the Ethics Committee of the University of Naples Federico II and was conducted in accordance with the Helsinki Declaration (Fortaleza revision, 2013), the standards of Good Clinical Practice (CPMP/ICH/135/95), and the pertinent European and Italian regulations on data protection. The NAFRA Project was registered on https://clinicaltrials.gov/ with the identifier NCT04719923. This study followed the Strengthening the reporting of observational studies in epidemiology (STROBE) guideline. The study design is graphically depicted in Fig. 1.

### Participants

Consecutively observed Caucasian patients of both sexes, aged from 18 months to 7 years, with a sure diagnosis of ASD (based on DSM-5 criteria) were considered eligible for the study [1]. The ASD children were classified as mild, moderate, or severe ASD level and of verbal or non-verbal type according to standard criteria [1].

Also NASD healthy controls visiting the Pediatric Surgery Center because of minimal surgical procedures (e.g., inguinal, or umbilical hernia, cryptorchidism, phimosis, labial fusion, pilonidal cyst, varicocele, moles, angioma) were evaluated for the study.

The exclusion criteria for both groups were: non-Caucasian ethnicity; age < 18 months or > 7 years; concomitant presence of epilepsy and other neurological diseases; diagnosis of chronic concomitant diseases (immunodeficiencies, type 1 diabetes and endocrine diseases, genetic and metabolic diseases, congenital heart diseases, autoimmune diseases, chronic infections, cystic fibrosis, chronic respiratory diseases (including asthma), chronic inflammatory bowel diseases, eosinophilic diseases of the gastrointestinal tract, functional gastrointestinal disorders, tumors); major malformations of the respiratory tract, gastrointestinal tract or urinary tract; previous gastrointestinal/urinary/lower respiratory tract/heart surgery; use of systemic antibiotics or anti-mycotic drugs or pre/pro/synbiotics during 12 weeks before study entry; investigator’s uncertainty about the willingness or ability of the subject to comply with the protocol requirements.

### Study outcomes

The main study outcome was the comparative evaluation of the presence of AFR (defined as the presence of at least one condition including food allergy, food intolerances or CD) in ASD pediatric patients vs. NASD controls.

Other outcomes were the comparative evaluation of nutritional status alterations (underweight, stunting, wasting, overweight and obesity), food selectivity and the MD-adherence in the two study groups.

### Sample size calculation

Based on our previous observational data, we expected a 16% incidence of AFR in patients with ASD. To detect a difference of at least 15% in the proportion of AFR between ASD patients and NASD healthy controls, and to account for a possible drop-out rate of up to 3%, we calculated that 100 subjects per group were needed at an alpha level of 0.05, with a power of 93% (Pearson’s chi-squared test) (Stata 14.0, Stata Corp, College Station, TX, US).

### Data collection

After the evaluation of eligibility, written informed consent was collected by all enrolled participants and their parents/legal guardian. Then, data regarding anamnestic and clinical features, gestational age, mode of delivery, birth weight, breastfeeding, breastfeeding duration, weaning age, maternal smoking during pregnancy, maternal antibiotic use during pregnancy, maternal pre-pregnancy body mass index (BMI), exposure to pets at home, living setting, parents/legal guardian educational/occupation level and socio-economic status, presence of siblings, familial allergic risk, familial risk of CD of all participants were also collected by the investigators in a dedicated clinical chart.

### Adverse food reactions assessment

All study subjects were assessed for the presence of any AFR through a careful evaluation of anamnestic and clinical features by the multidisciplinary team of the Tertiary Center of Pediatric Allergy, Gastroenterology and Nutrition. The Clinical Research Team was composed by pediatricians, pediatric allergists, pediatric gastroenterologists, dietitians/nutritionists, nurses, and biostatisticians. In subjects who had previously received a diagnosis of AFR, the robustness of the diagnosis was carefully evaluated by the Team. If any doubts regarding the previous AFR diagnosis emerged, these subjects underwent the diagnostic workup to confirm or rule out the AFR diagnosis. Similarly, all subjects with suggestive symptoms and signs of AFR, but without a sure diagnosis of AFR underwent an extensive diagnostic work up. Furthermore, the presence of previous and resolved AFR was also evaluated.

### Food allergy diagnosis

The food allergy (FA) diagnosis was obtained or confirmed using standard criteria (evaluation of anamnestic and clinical features, clear response to the elimination diet, screening tests results such as skin prick tests, serum specific IgE, atopy patch tests, and oral food challenge). The diagnostic work up was adopted in all study subjects reporting suggestive clinical features of FA (e.g., urticaria, angioedema, atopic dermatitis, diarrhea, regurgitation/vomiting, abdominal pain, constipation, bloody stools, failure to thrive after the ingestion of a specific food) [41, 42], following the diagnostic pathway for pediatric food allergies and intolerances in Italy proposed by the Italian Society for Pediatric Gastroenterology Hepatology and Nutrition (SIGENP) and the Italian Society for Pediatric Allergy and Immunology (SIAIP) [43].

### Assessment of food intolerances

The diagnosis of food intolerance or of CD was obtained or confirmed using standard criteria, including the evaluation of anamnestic and clinical features, the results of serologic and histologic tests, and of the hydrogen breath test. The diagnostic pathway was applied to participants presenting suggestive clinical features of food intolerances (stomach pain, bloating, gas/flatulence, diarrhea, rashes, urticaria, recurrent mouth ulcers or headaches, etc. after eating some foods) [43], following the diagnostic pathway for pediatric food allergies and intolerances in Italy proposed by the SIGENP and the SIAIP, and the European Society for Paediatric Gastroenterology, Hepatology and Nutrition (ESPGHAN) [43–45].

### Nutritional status alterations assessment

In order to assess the nutritional status alterations of all study subjects (underweight, stunting, wasting, overweight and obesity), experienced pediatric nurses and dietitians/nutritionists of the Clinical Research Team collected anthropometric parameters of all study subjects. Body weight and length/height were measured after 12-h fasting, with light indoor clothing and without shoes, using a calibrated mechanical column scale following standard procedures [46]. For children aged > 2 years, BMI was calculated as weight (in kilograms) divided by height (in meters) squared. The relative anthropometric parameters Z-scores were used to interpret growth measurements. Using the World Health Organization (WHO) growth charts of 2006 [47], nutritional status alterations were classified as: severe underweight (weight-for-age < − 3.00 Z-score), moderate underweight (weight-for-age from − 3.00 to -2.01 Z-score), severe stunting (length/height-for-age < − 3.00 Z-score), moderate stunting (length/height-for-age from − 3.00 to -2.01 Z-score), severe wasting (weight-for-length or BMI < − 3.00 Z-score), moderate wasting (weight-for-length or BMI from − 3.00 to -2.01 Z-score), overweight (weight-for-length or BMI from + 2.01 to + 3.00 Z-score up to 5 years of age; BMI from + 1.01 to + 2.00 Z-score from 5 to 19 years of age), and obesity (weight-for-length or BMI > + 3.00 Z-score up to 5 years of age; BMI > + 2.00 Z-score from 5 to 19 years of age).

### Assessment of food selectivity and mediterranean diet-adherence

The Food Preferences Inventory (FPI) was used to assess the food selectivity in all study subjects [48]. The FPI aims to investigate the variety of the diet. It is composed by a list, in which parents/caregivers report what food items of different groups (vegetables, fruits, dairy, carbohydrates, proteins, and miscellaneous food items) are typically consumed by their children, taking into account also what food items are usually offered to the entire family. Each accepted food items represent a score, whose sum reveal the total number of food items per group consumed by the children. A cut-off was obtained to define the selective phenotype. This cut-off represents the total score corresponding to the arithmetical mean minus 1SD of the accepted foods by the NASD children. Subjects reporting a score under the cut-off developed, were defined as selective [48, 49].

Finally, the KIDMED questionnaire, consisting of 16 questions aimed to assess the intake of key foods of MD, was used to assess MD-adherence. A total score ≤ 3 indicate poor MD-adherence, values ​​ranging from 4 to 7 indicate medium MD-adherence, and ≥ 8 indicate high adherence to MD [50].

### Statistical analysis

A clinical trial monitor reviewed the clinical forms for completeness, clarity, consistency, and accuracy. All the data were collected anonymously and entered into the study database using a single data entry method by the same researcher. The study database was cleaned according to standard procedures and was locked before statistical analysis by the statistical team. The Kolmogorov-Smirnov test was used to determine whether continuous variables were normally distributed, in which case they were reported as mean and standard deviation (SD). Continuous variables that were not normally distributed were reported as median and interquartile range (IQR) with minimum and maximum. Categorical variables were reported as the number and proportion of subjects with the characteristic of interest. The χ2 test and Fisher’s exact test were used for categorical variables. To evaluate the differences between continuous variables, the independent sample t test or Mann-Whitney U test were performed. Correlation analysis between KIDMED score and BMI was performed by Pearson correlation. The level of significance for all statistical tests was two-sided, *p* < 0.05. All analyses were performed using SPSS for Windows (SPSS Inc, version 23.0, Chicago, IL).

## Results

### Study subjects

From October 2017 to December 2023, 135 consecutive ASD patients were evaluated for the study. Of these, 6 subjects were excluded because the parents didn’t provide written informed consent for study participation, and 29 were excluded because the presence of exclusion criteria (11 for the presence of functional gastrointestinal disorders, 5 for recent antibiotic use, 3 for the presence of endocrine disorders, 2 for the presence of epilepsy, 2 for the presence of genetic conditions, 2 for the presence of congenital heart defects, 1 for a diagnosis of immunodeficiency, 1 for the presence of congenital heart defects, 1 for the presence of a malformation of the urinary tract, 1 for the presence of previous gastrointestinal tract surgery). Thus, a total of 100 ASD pediatric patients (mean ± SD age of 35.9 ± 7.4 months) were enrolled in the study. Regarding the type of ASD, 34% of patients had verbal type ASD and 66% had non-verbal type ASD; in addition, 16% of patients presented ASD level 1 (mild), 41% level 2 (moderate), and 43% level 3 (severe).

During the same study period, 116 NASD controls were evaluated for the study. 4 subjects were excluded for the lack of written informed consent by the parents, and 12 were excluded because the presence of exclusion criteria (5 for the presence of functional gastrointestinal disorders, 6 for a recent antibiotic use, 1 for the presence of congenital heart defect). So, a total of 100 NASD controls were enrolled in the study.

**Fig. 2:**
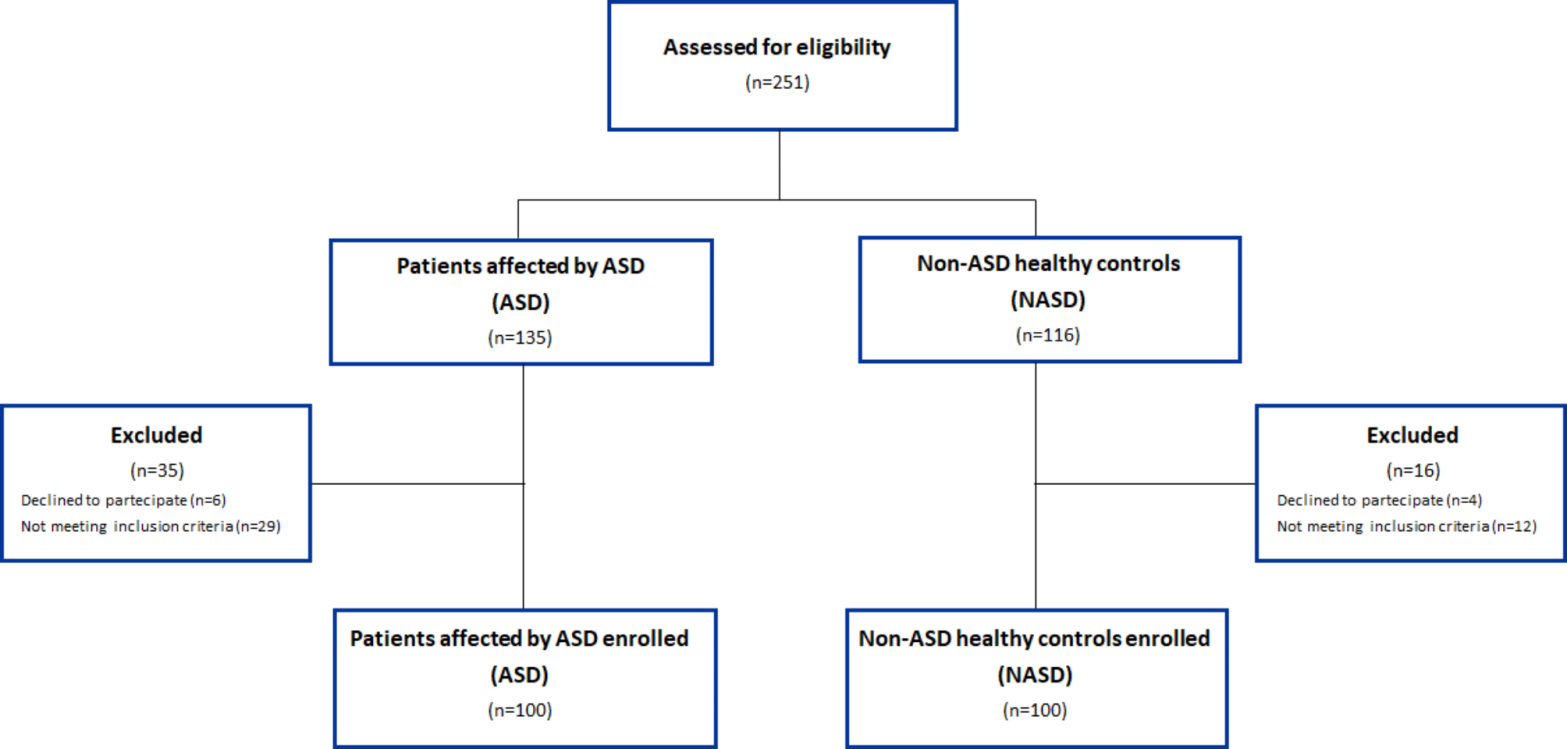
The flow of study subjects. Figure represents the flow diagram of participants of the NAFRA study

The flow of study subjects is depicted in Fig. 2.

The main anamnestic and clinical features of the study population are reported in the Table 1. Subjects enrolled in the two study groups were similar for the main demographic and anamnestic features.

**Table 1:** Main anamnestic and clinical features of the study population

	ASD (*n* = 100)	NASD (*n* = 100)
Male (%)	79 (79)	75 (75.2)
Age [months, (± SD)]	49.9 (15.4)	49.8 (17.7)
Spontaneous delivery (%)	38 (38)	32 (32)
Born at term (%)	87 (87)	78 (78)
Birth weight [kg, (± SD)]	3.21 (0.52)	3.13 (0.49)
Breastfeeding [at least 1 month, (%)]	61 (61)	73 (73)
Duration of breastfeeding [months, (mean ± SD)]	5.8 (5.4)	7.1 (7.1)
Weaning age [months, (± SD)]	5 (1)	4.9 (1)
Maternal smoking (%)	16 (16)	15 (15)
Maternal antibiotics use during pregnancy (%)	12 (12)	10 (10)
Maternal pre-pregnancy BMI (%) - Underweight (< 18.5 kg/m^2^) - Normal weight (18.5–24.9 kg/m^2^) - Overweight (25–29.9 kg/m^2^) - Obese (> 30 kg/m^2^)	2 (2) 64 (64) 24 (24) 10 (10)	1 (1) 71 (71) 21 (20) 7 (7)
Exposure to pets at home (%)	13 (13)	19 (19)
Urban setting (%)	97 (97)	81 (81)
Father’s level of education, (%) - Low (< 13 years of schooling) - Medium (13 years of schooling) - High (> 13 years of schooling)	40 (40) 38 (38) 22 (22)	43 (42) 39 (38) 18 (18)
Mother’s level of education, (%) - Low (< 13 years of schooling) - Medium (13 years of schooling) - High (> 13 years of schooling)	41 (41) 35 (35) 24 (24)	43 (42) 36 (36) 21 (21)
Father’s occupation level, (%) - Unemployed - Low - Medium - High	2 (2) 39 (39) 40 (40) 19 (19)	3 (3) 43 (42) 39 (38) 15 (15)
Mother’s occupation level, (%) - Unemployed - Low - Medium - High	31 (31) 14 (14) 39 (39) 16 (16)	29 (28) 15 (14) 41 (40) 15 (15)
Family socio-economic status, (%) - Low (8–27 points) - Medium (2–47 points) - High (48–66 points)	19 (19) 59 (59) 22 (22)	22 (22) 58 (58) 20 (20)
Presence of siblings (%)	53 (53)	67 (67)
Familial allergic risk [at least a first-degree allergic relative, (%)]	46 (46)	51 (50)
Familial risk of CD [at least a first-degree celiac relative, (%)]	2 (2)	3 (3)

*SD*,* standard deviations; BMI*,* body mass index; CD*,* celiac disease*

### Higher prevalence of AFR in ASD children

A significantly higher prevalence of AFR was observed in ASD patient compared with NASD subjects (16% vs. 2%, *p* = 0.001). Two out of 16 ASD patients presented with multiple conditions: one patient was diagnosed with both food intolerance and CD, while another subject had all three conditions (FA, food intolerance, and CD).

Eight ASD patients and 3 NASD controls, underwent a diagnostic workup at the Coordinating Center to confirm or obtain the FA diagnosis. After a careful evaluation by the multidisciplinary team, the FA diagnosis was obtained in 7 ASD patients and 1 NASD subject; the subject in the NASD group had multiple FA, while in the ASD group, 3 out of 7 subjects presented with multiple FA. Thus, the prevalence of FA was significantly higher in ASD patients if compared with NASD subjects (7% vs.1%, *p* = 0.03).

Among ASD patients diagnosed with FA, 57.1% (4/7) had IgE-mediated FA, while the remaining 42.8% (3/7) had a non-IgE-mediated form. No subject had outgrown FA.

The most common food antigen responsible for FA in ASD children was hen’s egg, affecting 71.4% of those with FA (all 4 patients affected by IgE-mediated FA and 1/3 patients affected by non-IgE-mediated FA). Cow’s milk was the second most common allergen, with 28.6% of ASD patients being allergic to cow’s milk proteins (1/4 patients affected by IgE-mediated FA and 1/3 patients affected by non-IgE-mediated FA). Additionally, 14.3% of ASD patients with FA had allergies to wheat (1/3 with non-IgE-mediated FA), soy (1/3 with non-IgE-mediated FA), legumes (1/4 with IgE-mediated FA), and fish (1/3 with non-IgE-mediated FA).

Regarding symptoms, 42.8% of ASD patients with FA exhibited cutaneous symptoms (2/4 patients with IgE-mediated FA and 1/3 patients with non-IgE-mediated FA), and the 71.4% presented with gastrointestinal symptoms (2/4 patients affected by IgE-mediated FA and 3/3 patients affected by non-IgE-mediated FA). The NASD control presented IgE-mediated form of FA to cow’s milk and hen’s egg, with cutaneous symptoms. Furthermore, neither of the subjects presented previous and resolved FA.

Additionally, six ASD patients underwent the diagnostic workup to confirm or obtain the CD diagnosis. After a careful evaluation by the multidisciplinary team the CD diagnosis was confirmed in all 6 ASD patients, with a significant higher prevalence if compared with NASD subjects (6% vs. 0%, *p* = 0.013).

Similarly, 6 ASD patients and 1 of NASD controls, underwent the diagnostic workup to confirm or obtain the diagnosis of food intolerance. Following evaluation by the multidisciplinary team, the diagnosis of food intolerance was confirmed in all 6 ASD patients, suggesting a trend toward a higher prevalence of this condition in ASD patients compared to NASD controls (6% vs.1%, *p* = 0.054). All subjects were affected by lactose intolerance. Supplementary Table 1 shows the number of subjects who underwent diagnostic workups at the Coordinating Center to confirm or establish a diagnosis of AFR, along with the tests that were performed. Supplementary Table 2 presents the number of subjects with AFR who received a confirmed diagnosis, categorized by type.

### Higher prevalence of nutritional status alterations in ASD children

In Table 2 are reported the median values of anthropometric parameters and relatives Z-scores of study participants. Significant differences were reported between the two study groups for the median values of body weight, weight-for-age Z-score, weight-for-length and BMI Z-scores, that resulted higher in ASD patients.

**Table 2:** Anthropometric parameters and relatives Z-scores of study participants

	ASD (*n* = 100)	NASD (*n* = 100)	*p*
**Body weight**,** median kg (IQR; min-max)**	18.5 (7; 13 and 55)	17 (7; 10 and 44)	*< 0.05*
**Weight-for-age Z-score**,** median (IQR; min-max)**	0.96 (1.8; -1.4 and 8.3)	0.07 (1.5; -2.6 and 6.5)	*< 0.05*
**Length/height**,** median cm (IQR; min-max)**	104.3 (17.3; 85.5 and 135)	103.3 (20.4; 75 and 134)	*ns*
**Length/height-for-age Z-score**,** median (IQR; min-max)**	-0.07 (1.6; -2.7 and 8)	-0.18 (2.2; -5.7 and 4)	*ns*
**Weight-for-length Z-score**, median (IQR; min-max)*	-0.63 (NA)**	0.31 (0.8; -0.05 and 1.6)	*< 0.05*
**BMI Z-score**,** median (IQR; min-max)**	1.41 (1.95; -3 and 10.0)	0.32 (1.8; 6.3 and 10.5)	*< 0.05*

*calculated for subjects ≤ 24 months at enrolment

** constant

*BMI*,* body mass index*

*Variables are reported as median (50th percentile) and interquartile range (IQR*,* 25th and 75th percentiles)*

Table 3 showed the prevalence in nutritional status alterations between the two study groups. No subjects presented severe underweight. A significant difference was observed in NASD controls, presented higher rate of moderate underweight compared to ASD patients (4% vs. 0% respectively, *p* < 0.05) and severe stunting (4% vs. 0% respectively, *p* < 0.05). A significantly higher prevalence of obesity was observed in ASD patients compared to NASD subjects (24% vs. 8% respectively, *p* < 0.05).

**Table 3:** Prevalence of nutritional status alterations between the two study groups

	ASD (*n* = 100)	NASD (*n* = 100)	*p*
**Severe underweight**,** n (%)**	0	0	*-*
**Moderate underweight**,** n (%)**	0	4 (4)	*< 0.05*
**Severe stunting**,** n (%)**	0	4 (4)	*< 0.05*
**Moderate stunting**,** n (%)**	4 (40)	10 (10)	*ns*
**Severe wasting**,** n (%)**	0	3 (3)	*ns*
**Moderate wasting**,** n (%)**	1 (1)	0	*ns*
**Overweight**,** n (%)**	11 (11)	11 (11)	*ns*
**Obesity**,** n (%)**	24 (24)	8 (8)	*< 0.05*

Discrete variables are reported as the number and proportion of subjects with the characteristic of interest

In ASD population, in patients with moderate ASD a higher prevalence of overweight (14.6%) and obesity (9.3%) was observed; similarly, in patients with severe form of ASD a higher prevalence of overweight (26.8%) and obesity (25.6%) was observed.

### Higher prevalence of food selectivity and lower MD-adherence in ASD children

A higher rate of food selectivity was observed in ASD patients than in NASD controls (26% vs. 2%, *p* < 0.001). The ASD patients also showed a general low adherence to the MD: 28% of ASD patients vs. 16% of NASD controls (*p = 0.041*) had a low MD-adherence; conversely, only 10% of ASD patients vs. 34% of NASD controls had a high MD-adherence (*p < 0.001*).

Analyzing the entire study population, 97.7% of subjects with high MD-adherence did not present overweight.

A significant negative correlation between KIDMED score and BMI was detected (*r*= -0.199, *p* = 0.006) in all study participants. In addition, the same negative correlation between KIDMED score and BMI was observed in ASD patients (*r*= -0.211, *p* = 0.037).

Finally, all NASD subjects with AFR also presented food selectivity.

## Discussion

The prevalence of ASD has been growing fast in the last decades worldwide. Appropriate multidisciplinary, comprehensive, and early approaches are required to provide tailored interventions, to support these patients, and to prevent and manage the comorbidities [3]. The AFR and the alterations in nutritional status have been reported as major comorbidities in ASD children but data are still conflicting. The NAFRA project was designed to fill this knowledge gap.

The NAFRA project was firstly launched to assess the AFR prevalence in ASD pediatric patients compared to NASD controls. Furthermore, nutritional status alterations, food selectivity and adherence to the MD were also evaluated. Lastly, the potential influence elicited by alterations of gut microbiome in the ASD pathogenesis is also under investigation in the same study population.

The results of the first clinical phase of the NAFRA project that we reported in this paper, confirm a higher rate of AFR in ASD patients compared to NASD controls. The higher rate of FA prevalence in ASD patients vs. NASD controls (7% vs. 1%) is well in line with what reported in previous studies based on parental-reported findings [9–13]. Similar results, based on physician documented diagnoses, were also reported in a case-control study in northern California, where data were recorded in medical records without specifying the diagnostic criteria or whether they were standardized [14]. This positive association between the co-existence of ASD and FA, reported in all published studies, was also confirmed in a recent meta-analysis including 12 published articles with 434.809 subjects [51]. In turn, in this meta-analysis it has been reported that the odds ratio of the ASD occurrence is 2.792 (95% CI: 2.081–3.746) for subjects affected by food hypersensitivity [51]. More recently, the results of a large retrospective study, involving 120.000 subjects with physician-made allergic disease diagnosis and 120.000 subjects without allergic disease, confirmed that allergic disorders could represent a risk factor for the occurrence of ASD [52].

Little is known about the association between food intolerances and ASD. Inaccurate and a specific diagnostic procedures were used in studies to evaluate the prevalence of food intolerances in ASD patients [15, 16]. To our knowledge, NAFRA is the first study aimed at confirming or diagnosing food intolerances according to accurate and standard criteria, and the results reported a trend toward higher food intolerances prevalence in ASD children than controls.

We also showed a significantly higher CD prevalence in ASD patients compared with NASD controls. To date, limited data exist on the possible association between CD and ASD [17]. In a well-designed Italian study evaluating a large number of ASD preschool children referred to a Tertiary Care University Hospital, the CD prevalence resulted significantly higher than the general pediatric population not affected by ASD [18]. Conversely, other studies reported a weak association between CD and ASD [19–21]. The low number of subjects enrolled, the non-matching for sex between case and controls that generates selection bias, and the changes in the diagnostic approach and guidelines for CD during the years, could represent the main limitations of these findings. Lastly, several studies and meta-analysis suggested that CD could be a risk factor for the developing of ASD [53–55].

Gut microbiome alterations could represent the underlying mechanisms. In ASD patients, the gut microbiome dysbiosis has emerged as a significant area of research interest, prompting researchers to examine the gut microbial features of these patients, but little is known about the connection and contribution of gut microbiome alterations to the pathophysiology of AFR in autistic patients [56–59]. Dietary factors, including the nutritional components of foods (carbohydrates, fats, proteins, vitamins, minerals, etc.), food additives, cooking and processing methods of food, impact the gut microbiome structure and function, and these are closely related to the AFR susceptibility, by modulating intestinal barrier permeability, type 2 immunity, and inflammation [60–62]. Shaping the gut microbiome diversity and features, dietary habits could represent the main environmental factor that can modulate immune system function, inflammatory disease, energy homeostasis etc. Thus, unhealthy dietary habits may lead to the double burden of immune-mediated/inflammatory diseases such as AFR and of nutritional status alterations, potentially related to gut microbiome alterations [39, 40, 63, 64].

A growing body of evidence has shown that ASD children have atypical dietary habits if compared to their peers typically developing. As we expected, and in accordance with previous findings, also in this study we have showed that ASD children presented food selectivity and low adherence to the MD if compared with non-autistic controls [25, 29, 36, 38].

Much has changed regarding the effects of the typical dietary habits of ASD patients on their nutritional status. Previous evidence reported that ASD patients could present nutritional deficiencies and undernutrition due to food selectivity, however a growing body of literature has shown that children with ASD are characterized by obesity and excess of some nutrients [39]. In this study we observed a significantly higher rate of obesity among ASD patients, such as the main form of nutritional status alterations, and this result is well in line with other investigations. Indeed, several studies, systematic reviews and meta-analyses performed to analyze the weight status of ASD children, reported significantly higher prevalence of obesity among ASD patients if compared to non-autistic counterparts [22–26]. Furthermore, it has been reported that ASD condition seem to increase the risk of childhood obesity, representing a risk factor for the disease [65]. One significant contributor could be the food preferences, which lead to selective eating habits and a limited range of food choices, often favouring calorie-dense or ultra-processed foods. Additionally, challenges with social communication and interaction might hinder participation in physical activities or sports, reducing opportunities for regular exercise. Furthermore, difficulties in understanding and responding to hunger cues or regulating emotions may lead to overeating or seeking comfort in food [36, 66]. A multidisciplinary parent training program that involves nurses, dietitians/nutritionists, and physicians has shown to be effective in improving dietary habits and reducing disruptive mealtime behaviours. This holistic approach not only increases the variety of foods accepted by children but also supports parents in managing feeding issues more effectively, contributing to overall better health outcomes for children with ASD [67].

This study presents several strengths. The multidisciplinarity of the team, including pediatricians, pediatric neuropsychiatrists, nurses, dietitians/nutritionists, and allergists, ensures a rigorous assessment of the study subjects, while the rigorous methodology enhance the study’s reliability. Notably, the findings of higher rates of AFR, obesity, and poor dietary habits in ASD children underscore the critical need for tailored nutritional interventions.

However, the study has also limitations. The cross-sectional design restricts the ability to draw causal inferences, and the relatively small sample size may limit the generalizability of the results. Additionally, conducting the study at a single center and potential selection bias could affect the representativeness of the findings. Despite these limitations, the NAFRA project significantly contributes to our understanding of the nutritional challenges faced by children with ASD and highlights the importance of early and comprehensive dietary management.

## Conclusion

The high rate of AFR, obesity and unhealthy dietary habits in ASD children, pointed out in the NAFRA project, strongly suggest the importance of a multidisciplinary approach, providing early and appropriate nutritional management to improve core and associated ASD-related conditions. This multidisciplinary approach through tailored interventions could have a pivotal role in the early phase of ASD management for preventing and treating relevant co-morbidities in these children, and to improve their quality of life.

## Electronic supplementary material

Below is the link to the electronic supplementary material.

Supplementary Material 1

Supplementary Material 2

## Data Availability

The dataset used and/or analyzed during the current study is available from the corresponding author on reasonable request.

## Abbreviations

ASD: Autism Spectrum Disorder
AFR: Adverse food reactions
CD: Celiac disease (CD)
MD: Mediterranean Diet (MD)
NAFRA: Nutritional status and Adverse Food Reactions in children with Autism Spectrum Disorder)
NASD: Non-ASD healthy controls
BMI: Body mass index
FA: Food allergy
SIGENP: Italian Society for Pediatric Gastroenterology Hepatology and Nutrition
SIAIP: And the Italian Society for Pediatric Allergy and Immunology
ESPGHAN: European Society for Paediatric Gastroenterology, Hepatology and Nutrition
WHO: World Health Organization
FPI: Food Preferences Inventory
SD: Standard deviation
IQR: Interquartile range
