# Supplementary material for: Adverse food reactions and alterations in nutritional status in children with autism spectrum disorders: results of the NAFRA project
Source: Ital J Pediatr. 2024 Nov 4;50:228. doi: 10.1186/s13052-024-01794-8 (PMC11533279; doi:10.1186/s13052-024-01794-8)
Supplement: Supplementary file 1 — Supplementary Material 1 [file 13052_2024_1794_MOESM1_ESM.docx]

Naples, September 27^th^ 2024

Dear Prof. Carlo Caffarelli,

Editor of *Italian Journal of Pediatrics*

We are delighted to learn that our paper entitled “Adverse Food Reactions and Alterations in Nutritional Status in Children with Autism Spectrum Disorders: results of the NAFRA project" (Manuscript Number: ITJP-D-24-00669R1) could be published in your prestigious Journal.

Enclosed please find the revised version of the above-mentioned manuscript (clean version of the revised manuscript and version with tracked changes), in which we have addressed all the remarks made by Reviewers (see point-by-point reply enclosed herewith).

We are grateful to the Reviewers for the supportive comments, and we feel that thanks to these precious suggestions the paper is now greatly improved. We hope you will now find it suitable for publication in *Italian Journal of Pediatrics*,

Looking forward to a fast, and hopefully, positive reply, I am,

Warm regards,

Rita Nocerino

Dr. Rita Nocerino, RN, MSN, PhDs

Department of Translational Medical Science

ImmunoNutritionLab at CEINGE – Advanced Biotechnologies

Department of Biomedicine and Prevention, University of Rome “Tor Vergata”, Rome, Italy

University of Naples “Federico II”

Via S. Pansini 5 80131 Naples, Italy

P.: +390817464542

E-mail: [rita.nocerino@unina.it](mailto:rita.nocerino@unina.it)

ORCID ID: <https://orcid.org/0000-0003-4681-546X>

**Reviewer #1: The authors responded with corrections and integrations to my considerations. However, I still have 2 comments to make about these integrations
Line 12: A significantly higher prevalence of AFR was observed in ASD patient compared with NASD 12  subjects (16% vs. 2%, p=0.001).   If we are talking about the children enrolled in the study (100) there should therefore be 16 children with AFR. However FA diagnosis was obtained in 7, six obtained diagnosis of Celiac Disease and other 6 diagnosis of lactose intolerance: 7 + 6 + 6 = 20 and not 16. I don't understand. Why don't the numbers coincide?  It is a typo? Did the same child have 2 diseases? It is necessary to explain why**

*We thank the Reviewer for this comment. The reported prevalence is accurate, as 2 out of 16 ASD patients presented with multiple conditions: one patient was diagnosed with both food intolerance and celiac disease, while another subject had all three conditions (food allergy, food intolerance, and celiac disease). However, we have modified the text to better clarify this point.*

**Lina 18: Among ASD patients diagnosed with FA, 57.1% (4/7) had IgE-mediated FA, while the remaining 42.8% (3/7) had a non IgE-mediated form.   Supplementary Table 1 shows that 8/8 had both specific IgE and SPT positive: what non-IgE-mediated allergy does a child with specific IgE and SPT positive have? It is a typo? It is necessary to explain the inconsistency of the data.**

*Supplementary Table 1 lists the number of subjects who underwent diagnostic workups at the Coordinating Center to confirm or establish a diagnosis of AFR, as well as the diagnostic tests that were performed. However, the table does not indicate the positivity of the tests.
It is important to note that SPT and serum IgE tests were conducted on all subjects with suspected FA.*

*The atopy patch tests were performed in the 3 subjects who had negative SPT and serum IgE results. In all these subjects a diagnosis of non-IgE-mediated FA was confirmed.*

*The legend of the Supplementary Table 1 was changed according to this point.*

**Reviewer #2: I thank the AA for their responses to my comments and the changes they have made.

I think, however, that there was a misunderstanding because I had asked for a table with detailed data both for the 100 patients in the ASD group and for the 100 in the control group, instead of the aggregated data.
In this table, the frequency of the various diagnostic criteria, both clinical (e.g. anaphylaxis, urticaria, angio-oedema, atopic dermatitis, diarrhea, regurgitation/vomiting, abdominal pain, constipation, bloody stools, failure to thrive after ingestion of a specific foodstuff) and immunological (SPT, specific IgE, CRD) must be reported in numerical values and %. If possible separate for the main foods found to be allergenic: egg, milk, wheat and a 4th column for other foods.
A second table with similar criteria should be prepared to describe the two populations for food intolerance with both clinical and laboratory data (Hydrogen breath test)


Consequently, the supplementary table 1 of number of patients that underwent a diagnostic workup of AFR (first part of the table) can be removed. While the second part of the same table can be left specifying that the results refer to patients who have undergone full diagnostic workup.**

*In response to the previous request provided by the Reviewer,* **'It is not reported how many and which tests were performed in the active and control group. A table with a detailed description of the various diagnostic tests (e.g., only clinical history, SPT/IgE/OFC) for the two groups must be provided,'** *we have included the Supplementary Table 1. This Table lists the number (and %) of subjects from each group who underwent diagnostic workups at the Coordinating Center to confirm a previous diagnosis or to obtain a new diagnosis of AFR, as well as the tests that were performed. As mentioned in the text, the diagnostic workup was not conducted on all 100 subjects in each group, but rather on those in both the ASD and NASD groups who had previously received a diagnosis of AFR, or where doubts arose regarding the accuracy of a previous AFR diagnosis. The data in Supplementary Table 1 therefore reflect these specific subjects.*

*However, according to the new Reviewer comment, in the revised version of the Table we now also reported the allergenic food for each group, and for the immune mechanism of FA.*
